# Plasma Treatment Effects on Oral *Candida albicans* Biofilms

**DOI:** 10.31487/j.dobcr.2021.02.05

**Published:** 2021-06-01

**Authors:** Qing Hong, Xiaoqing Dong, Meng Chen, Hongmin Sun, Liang Hong, Yong Wang, Qingsong Yu

**Affiliations:** 1 Department of Mechanical and Aerospace Engineering, University of Missouri, Columbia, Missouri, USA; 2 Nanova, Inc., Columbia, Missouri, USA; 3 Department of Internal Medicine, University of Missouri, Columbia, Missouri, USA; 4 Department of Pediatric and Community Dentistry, College of Dentistry, University of Tennessee Health Science Center, Memphis, Tennessee, USA; 5 Center for Research on Interfacial Structure & Properties, School of Dentistry, University of Missouri-Kansas City, Kansas City, Missouri, USA

**Keywords:** Low-temperature plasma, oral fungal biofilm, biofilm disinfection, Candida albicans

## Abstract

The objective of this study is to evaluate the plasma treatment effects on oral fungal biofilms. *Candida albicans* biofilms were developed on the 48-well plate to serve as a model of oral fungal biofilm. The treatment of 0.2% chlorhexidine digluconate (CHX) was used as a positive control compared with plasma treatments. The efficacy of treatments was determined by 3-(4,5-dimethylazol-2-yl)-2,5-diphenyl-2H-tetrazolium bromide (MTT) assay and confocal laser scanning microscope (CLSM). The survival percentage of *Candida albicans* decreased from 52% to 27% as the plasma power increased from 6mA to 8mA and plasma exposure time extended from 2 min to 10 min. Moreover, it was found that there is a synergistic effect of the combination of plasma and CHX treatments. Scanning electron microscopy (SEM) examination indicated severe cell damages resulting from plasma treatment. In conclusion, the low-temperature plasma treatment is effective in deactivating *Candida albicans* biofilms and thus provides a promising alternative to disinfect oral fungal biofilms.

## Introduction

*Candida albicans* (*C. albicans*), an asexual diploid fungus, is one of the organisms that prefer human mouths as their primary habitat. It is an oral commensal bacterium and can be found in 40% of normal subjects and 75% of patients who wear a denture [1]. *C. albicans* has the ability to morph from a free-floating form to a biofilm form that grows as a community. Biofilm may allow for overpopulation and nutrient load control. The formation of biofilm requires *C. albicans* to grow hyphae which are multinucleated cell filaments [2]. The hyphae could help *C. albicans* survive under unfavourable conditions such as increased temperature, increased pH levels, nutrient starvation and increased cell density [3]. Numerous studies have reported that there is an association between *C. albicans* and denture-related stomatitis which is termed as *Candida*-associated denture stomatitis [4, 5]. The characteristic presenting features of denture stomatitis are chronic erythema and inflamed mucosa, particularly under the upper denture. The patients may complain a burning sensation, discomfort, or bad taste [6, 7].

A number of antifungal agents are available on the market. The most popular antifungals used against *Candida* infection are polyenes (such as amphotericin B and nystatin) and azoles (such as fluconazole and itraconazole) [5, 8, 9]. However, the increased use of antifungal agents in recent years has resulted in the development of resistance to these drugs [10–13]. Besides these two types of drugs, chlorhexidine, contained in mouth rinses, is also an appropriate option to conventional antifungals in the management of oral candidiasis [14]. A common side effect associated with chlorhexidine oral rinses is an increase in staining of teeth and other oral surfaces. Gas plasma, described as the fourth fundamental state of matter, consists of many active plasma species/particles including positively charged ions and negatively charged electrons, electronically excited neutral atoms and molecules, radical, and ultraviolet (UV) photons [15]. These active particles are responsible for the antimicrobial efficacy of plasma [16]. Low-temperature plasma generated at atmospheric pressure becomes a potential tool for oral microbial decontamination [16–19]. Previous studies have demonstrated the low temperature plasma was capable of deactivating planktonic cavity-causing bacteria [20–24]. Several other studies have confirmed the antimicrobial capability for *Streptococcus mutans* biofilms, which are the most significant cariogenic microorganism [5, 25–28]. These works encourage the application of low-temperature atmospheric plasma on disinfection of oral candidiasis, which is a yeast/fungi infection of the genus *Candida* that develops on the mucous membranes of the mouth. In this paper, plasma treatment effects on oral *Candida albicans* biofilms were studied *in vitro*.

## Materials and Methods

### I The Low-Temperature Atmospheric Plasma Brush

**Figure 1:**
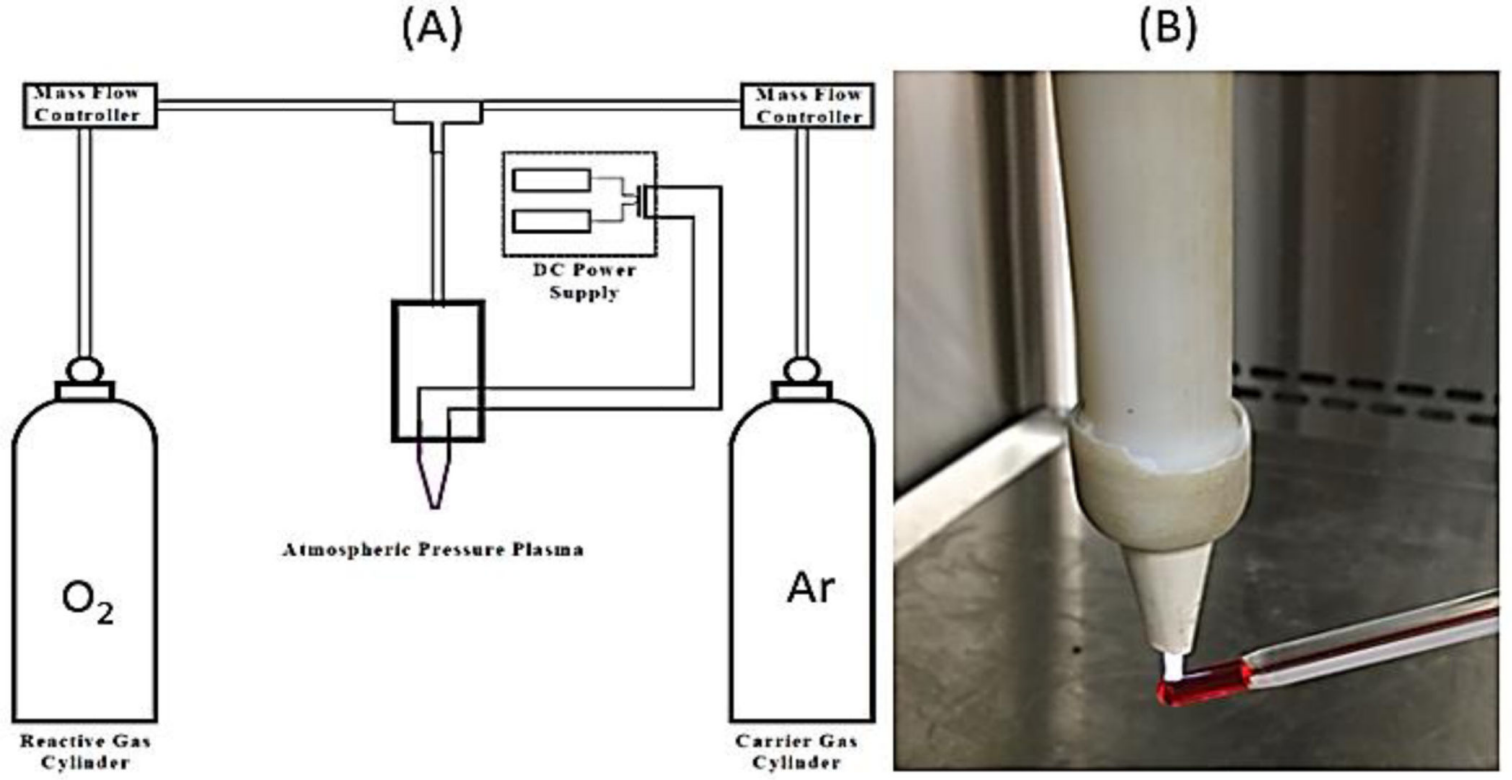
**A)** Schematic of plasma brush system and **B)** a photograph of temperature measurement of the plasma flame.

A low-temperature atmospheric plasma brush was employed in this study. The plasma brush is composed of a gas source, a power supply, and a discharge chamber, as shown in (Figure 1A). The gas, argon and oxygen, pass through the discharge chamber at a controlled flow rate. An electrical field was applied to the two electrodes located inside the chamber to ignite a DC glow discharge by a DC power supply (Spellman SL60, New York, USA). The power input in generating and sustaining DC atmospheric pressure plasma is at the level of several watts or tens of watts. The low power input of this plasma system provides a unique advantage in the low power consumption. The physical properties of the system have been described in the other works [22, 29]. A thermometer (VWR 61066–104, Chicago, IL) was used to measure the plasma temperature. The ignited plasma touched the tip of the thermometer, as seen in (Figure 1B). The temperature was recorded every 20 seconds and the starting temperature was the beginning of plasma ignition. The plasma was operated at 6mA and 8mA with 3000 standard cubic centimeters per minute (sccm) of argon and 30 sccm of oxygen.

### II Biofilm Preparation

One milliliter of frozen-stored *C. albicans* (ATCC 18804) was defrosted for 15 minutes and cultured overnight in 25mL Sabouraud (SAB) liquid medium (Fisher Science, Pittsburgh, PA, USA). The sub-cultured *C. albicans* suspension was diluted to the density of 1×10^7^ cells/mL. Then 600μL of diluted *C. albicans* suspension was added into each well of the 48-well plate and incubated in an incubator for 24 hours at 37°C.

### III Plasma Treatment

As the biofilms were well developed, the SAB medium was removed from the wells by gentle aspiration. The biofilms were washed twice with phosphate-buffered saline (PBS) to remove non-adherent bacterial cells. Before any disinfection treatment, biofilms were dried in the hood for 5 minutes. All prepared biofilms were divided into 8 groups to receive different treatments, as seen in (Table 1). One group of prepared biofilms was treated with 150μL of 0.2% chlorhexidine digluconate (CHX) solution (Sigma Aldrich, St. Louis, MO) for ten minutes. Another six groups of biofilms were treated with selected plasma conditions. The six different plasma conditions applied in this study were 6mA power supply treated for 2, 5 or 10 minutes, and 8mA power supply treated for 2, 5 or 10 minutes. The gas flow was 3000 sccm of argon with 30 sccm of oxygen. In addition, one group without any disinfection treatment was regarded as a control group. Each group contained five samples. In order to investigate the synergistic effect of plasma and CHX treatment on the fungal biofilms, the prepared biofilms were first treated by plasma as described above and followed by CHX treatments [Group 9 and Group 10 shown in (Table 1)]. Five samples were tested for each group.

### IV MTT Assay

Cell proliferation kit I (Roche Diagnostics, Fisher Scientific, Pittsburgh, PA, USA) was used for the 3-(4, 5-dimethylazol-2-yl)-2,5-diphenyl-2H-tetrazolium bromide (MTT) assay. This kit includes MTT label reagent and solubilization (check the name) solution. After disinfection treatments, 500μL PBS was added to each well and blown with a pipette for 10 times to detach the bacterial cells from the well substrate. The 500μL PBS suspension containing bacterial cells was transferred into a 1.5mL centrifuge tube and centrifuged at 6000 rpm for eight minutes. After centrifugation, PBS was removed, and cell bullet was kept in the tube. 200μL fresh PBS was added into the tube to suspend cells on a vortexer for 30 seconds. The cell suspension was mixed with 20μL of the MTT label reagent in the tube and incubated for four hours at 37°C. Afterwards, 200μL of the solubilization solution in the kit was added to the tube. The tubes were put into an incubator overnight. Then, 100μL of the solution was transferred to a 96-well plate for optical density assay at 570 nm. The bacterial survival percentage was calculated against the mean of biofilms on control samples. The experiment was performed three times to obtain means and standard errors of means.

### V Confocal Laser Scanning Microscope (CLSM) Analysis

In order to conduct CLSM measurement, *C. albicans* biofilms were developed on polystyrene strips and treated by following the procedure in (Table 1). The strips were cut from Petri dishes (Fisher Scientific, Pittsburgh, PA 15275, USA). After treatments, the adherent biofilms were stained using a Live/Dead BacLight bacterial viability kit (Invitrogen, Carlsbad, CA), which includes propidium iodide (PI) and SYTO9. In the experiment, SYTO9 and PI were mixed together according to the manufacturer’s instructions. 0.5mL of the mixture was dropped on the biofilm surface and then incubated in the dark for 15 minutes in a hood. After removing the staining medium, the biofilms were immersed in a 10% formalin solution for 30 minutes in order to fix the structure of biofilm. Then, the samples were stored in chambered cover glasses with PBS until CLSM analysis. The biofilms were visualized with a confocal laser scanning microscope (Zeiss LSM 510, Carl Zeiss Micro-Imaging GmbH, Jena, Germany). 3-Dimensional structural reconstruction of CLSM data was performed using Imaris 4.0 (Bitplane AG, Zurich, Switzerland).

### VI Scanning Electronic Microscope (SEM) Examination

The treated and untreated biofilms were prepared as described in (Table 1). Samples were fixed with 2% glutaraldehyde- 2% paraformaldehyde in 0.1 M cacodylate buffer (pH=7.4) for 2 hours at 4°C and subsequently fixed with 0.1% osmium tetroxide for 1 hour. After being washed in ultrapure distilled water, the biofilms were dehydrated in graded ethanol series (20%, 50%, 70%, 90%, 100%, 100% and 100%) for 15 min each. After critical point drying and platinum sputter coating, samples were examined by using a scanning electron microscope (FEI Quanta 600FEG).

### VII Statistical Analyses

For experimental data evaluation, the Sigma statistical analysis programme was used. The comparisons of different treatment groups were performed by using the one-way analysis of variance (ANOVA), and differences with a P value less than 0.05 (p < 0.05) among the groups were considered to be statistically significant.

## Results

### I Plasma Temperature

**Figure 2:**
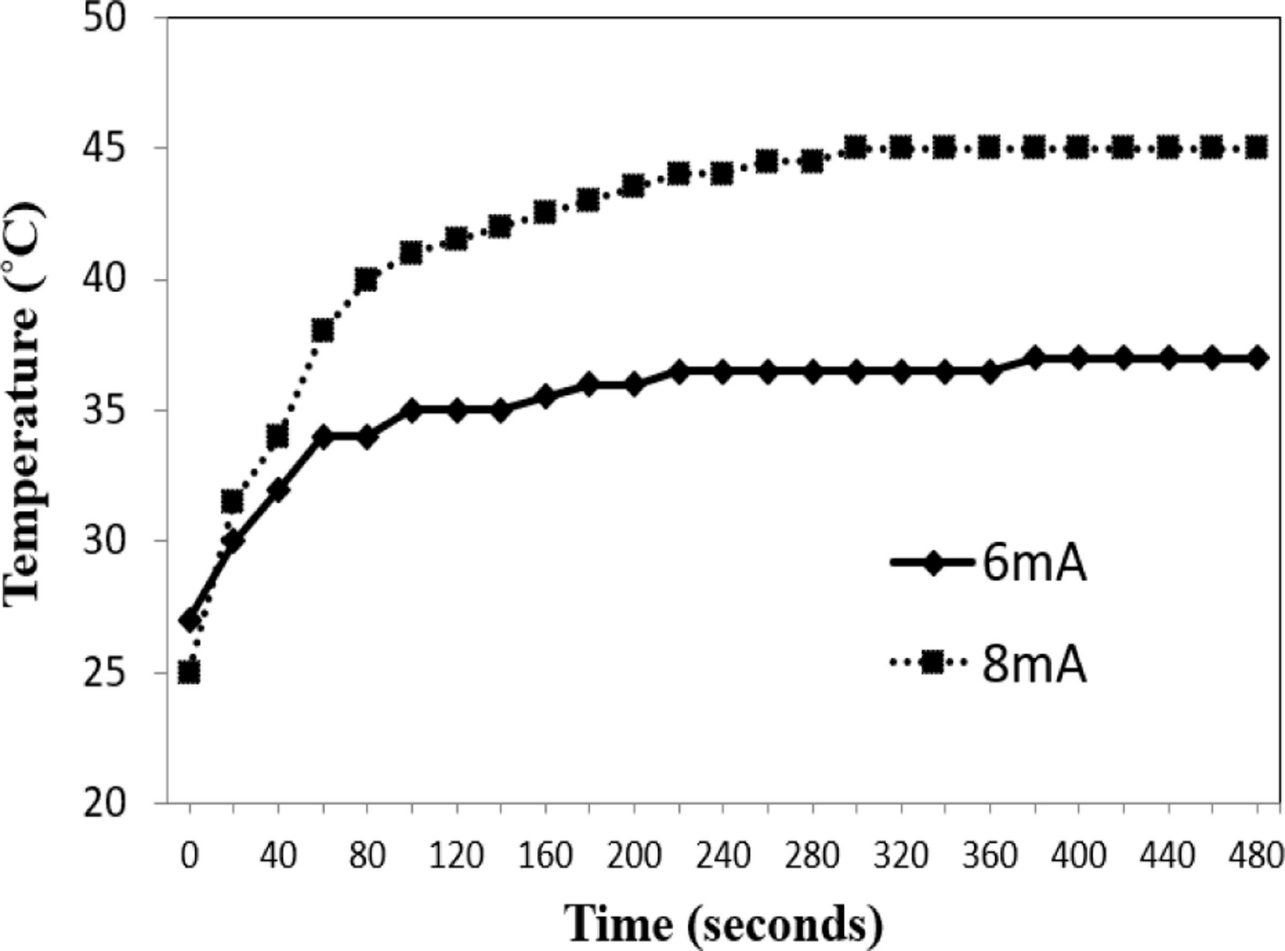
Time dependence of plasma flame temperature of the low-temperature atmospheric plasma brush operated under 6mA and 8mA, respectively.

The plasma temperature was measured with a thermal meter, as shown in (Figure 2). At 6mA, the plasma temperature rises sharply from 27°C to 34°C during the first 60 seconds. Afterwards, the temperature gently increases to 35°C in the next 300 seconds. Eventually, the temperature stays at 35°C till the plasma brush is turned off. In contrast, the temperature at 8mA presents a similar trend but different peak temperature. At 8mA, the plasma temperature rises sharply from 24°C to 40°C during the first 80 seconds. Afterwards, the temperature gently increases to 45°C in the next 200 seconds. Eventually, the temperature stays at 45°C till the plasma brush is turned off. The peak temperature at 8mA is 10°C higher than that at 6mA.

### II Disinfection Effect of Plasma Treatment

**Figure 3:**
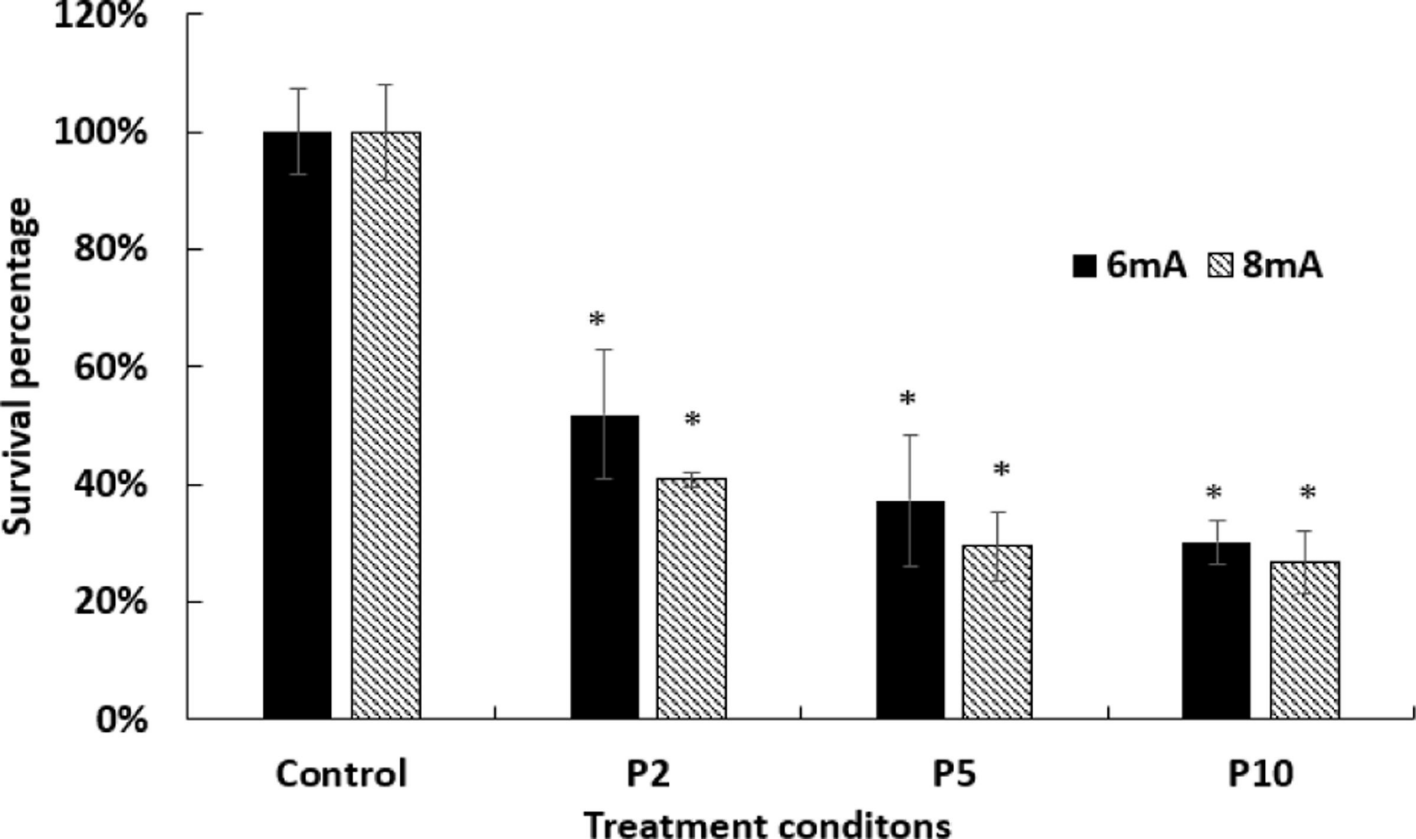
Plasma treatment time dependence of *C. albicans* biofilm survival percentage. Control: untreated. P2: 2-minute plasma treatment. P5: 5-minute plasma treatment. P10: 10-minute plasma treatment. The asterisk shows significant differences between different conditions and the control group (p < 0.05).

Biofilms were plasma treated for 2, 5 and 10 minutes under 6mA or 8mA power supply, respectively. MTT assay was performed to measure the disinfection efficiency of plasma treatment on the *C. albicans* biofilm. This is a widely used assay to measure bacterial viability. A significant reduction of fungal survival rate was observed on all plasma treatment groups as compared to the untreated control group shown in (Figure 3). Furthermore, the disinfection efficiency increased with plasma treatment time. At 6mA, the fungal survival rates were 52% for 2 min plasma treatment, 37% for 5 min plasma treatment, and 30% for 10 min plasma treatment, respectively. Likewise, at 8mA, the fungal survival rates were 32% for 2 min plasma treatment, 30% for 5 min plasma treatment and 27% for 10 min plasma treatment, respectively. In addition, a higher plasma power could provide better disinfection efficiency, especially for short plasma treatment time. The survival rate under 8mA was 10% less than that under 6mA for 2 min plasma treatment, while there were only 7% and 3% differences between 8mA and 6mA as the plasma treatment time extends from 5 min to 10 min.

### III Synergistic Effects of Plasma Treatment with CHX against *C. albicans* Biofilms

**Figure 4:**
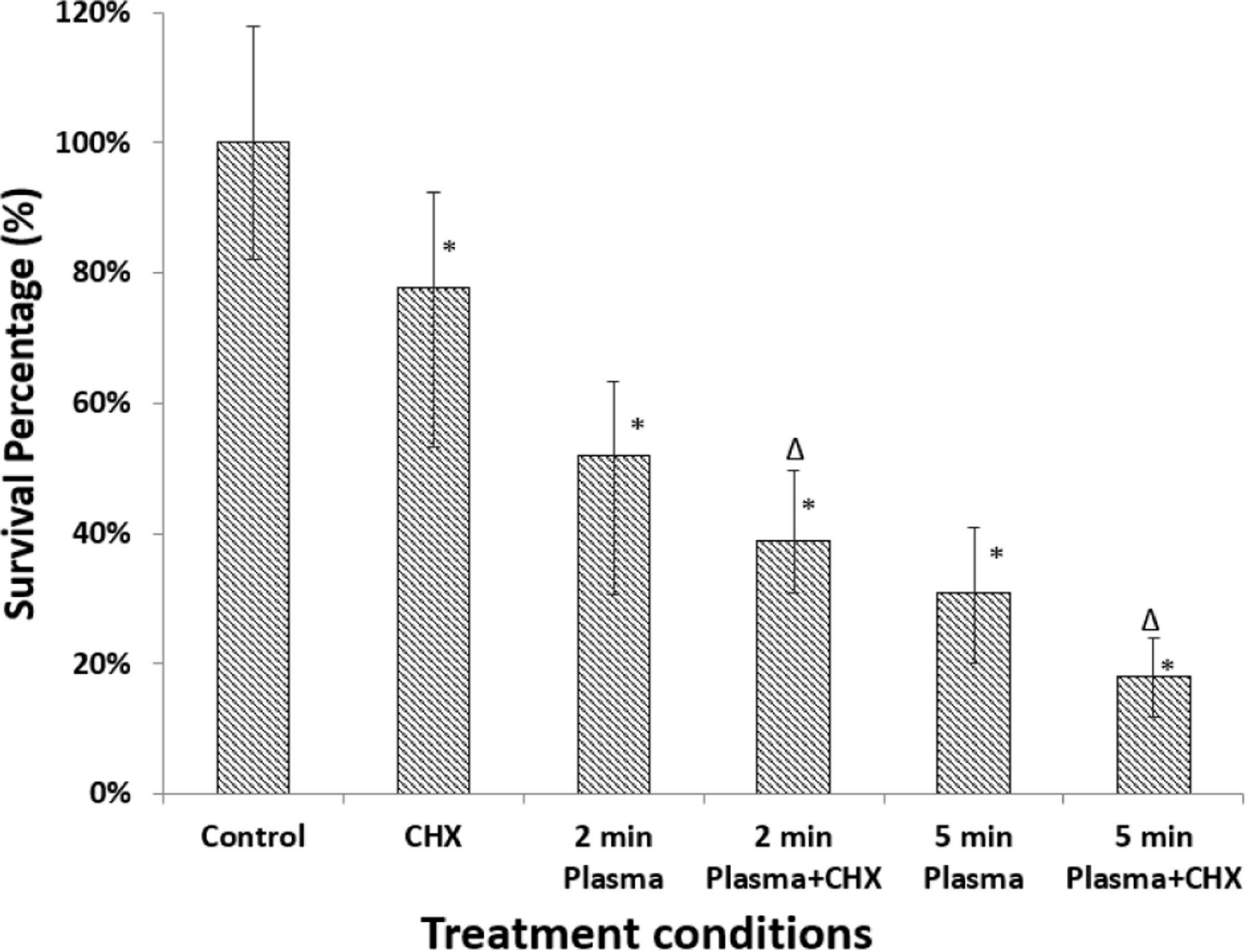
Biofilm survival percentage with different plasma and CHX treatments. The asterisk shows significant differences between the different treatment conditions and the control group (p < 0.05). The triangle shows significant differences between the single plasma treatments and the combination of plasma and CHX treatments.

Figure 4 shows the fungal survival rate of the biofilms with CHX treatment only and plasma treatment subsequently followed by CHX treatment. It can be seen that CHX treatment has a certain capability of inactivating *C. albicans* in the biofilms. With 10 min CHX treatment time, the survival rate was 77% compared with the untreated control group. In contrast, the plasma treatments subsequently followed by the CHX treatment became much more effective in fungal deactivation. CHX treatment on the 2 min plasma-treated biofilms induced as low as 39% fungal survival rate. Likewise, CHX treatment on the 5 min plasma treated biofilms had even lower 18% fungal survival rate. The combination of plasma treatment and CHX treatment also offered better deactivation effects than single plasma treatment. The single 2 min plasma treatment presented 52% survival rate and the single 5 min plasma treatment presented 31% survival rate, as seen in (Figure 4).

**Figure 5:**
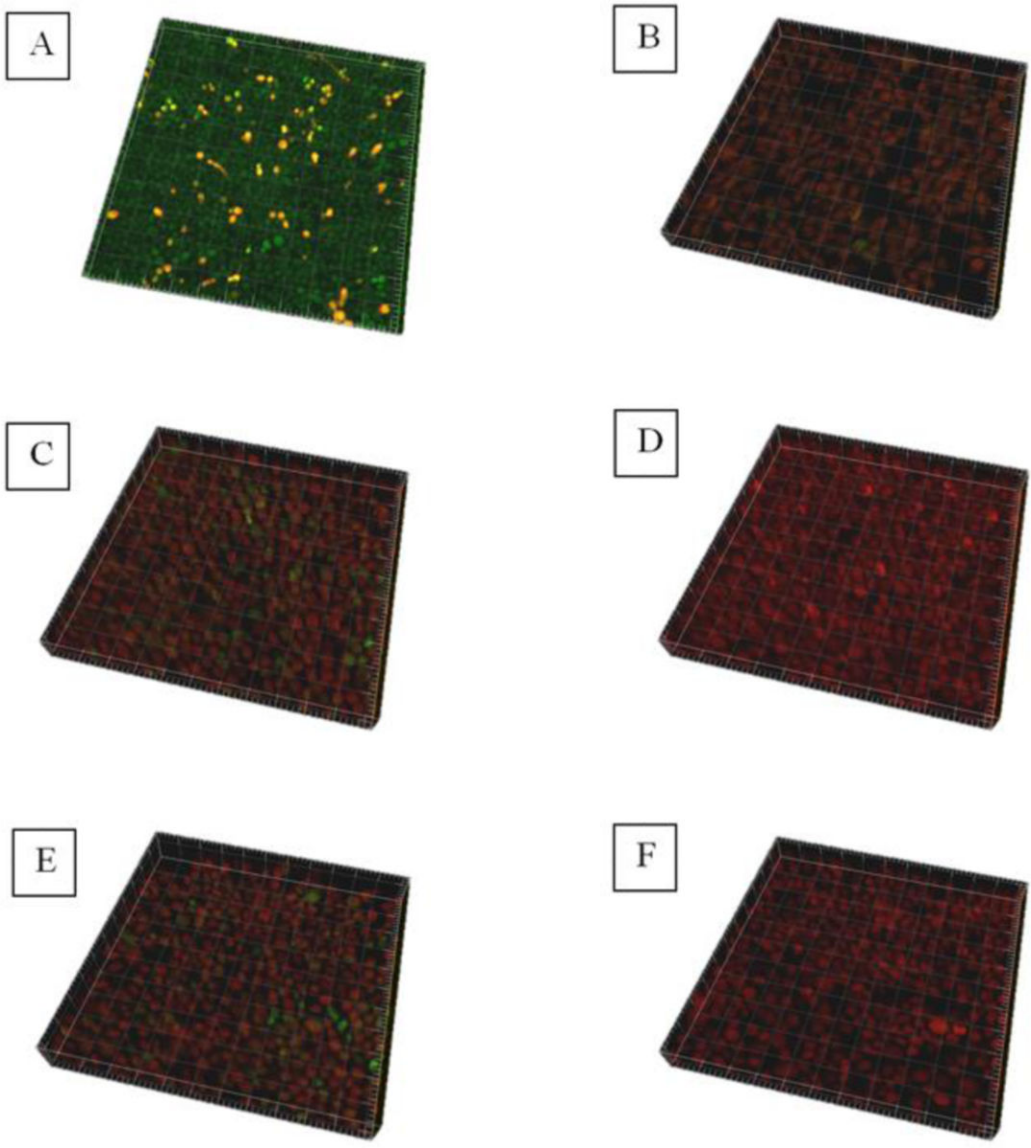
CLSM images of **A)** Untreated Control; **B)** CHX treatment; **C)** 5 min treatment by 6mA plasmas; **D)** 2 min treatment by 6mA plasmas + CHX treatment; **E)** 5 min treatment by 8mA plasmas; **F)** 5 min treatment by 8mA plasmas + CHX treatment. Green dots indicate the living fungal cells and red dots indicate the dead fungal cells.

The confocal laser scanning microscope (CLSM) with Live/Dead BacLight method was applied to examine the fungal viability in the biofilms. According to the mechanism of Live/Dead BacLight dyes, the viable fungi would be stained with SYTO9 and emit a green fluorescence, whereas dead fungi would be stained with PI and emit a red fluorescence [30]. As seen in (Figure 5), multilayer biofilms were formed on polystyrene strips. Most fungi on the untreated control samples (Figure 5A) were green, while a majority of fungi on other samples emitted red fluorescence. In other words, all of the treatments used in this experiment were capable of deactivating fungi in biofilms. Moreover, several green dots could be observed in the groups of CHX treatment (Figure 5B), 6mA plasma treatment for 2 min (Figure 5C) and 8mA plasma treatment for 5 min (Figure 5E). However, there are no green dots presented in the samples with combinations of plasma treatment and CHX treatment, as seen in (Figures 5D & 5F). In other words, combined treatments using plasma and CHX were more efficient in deactivating *C. albicans* biofilms than both the single CHX treatment and single plasma treatment.

**Figure 6:**
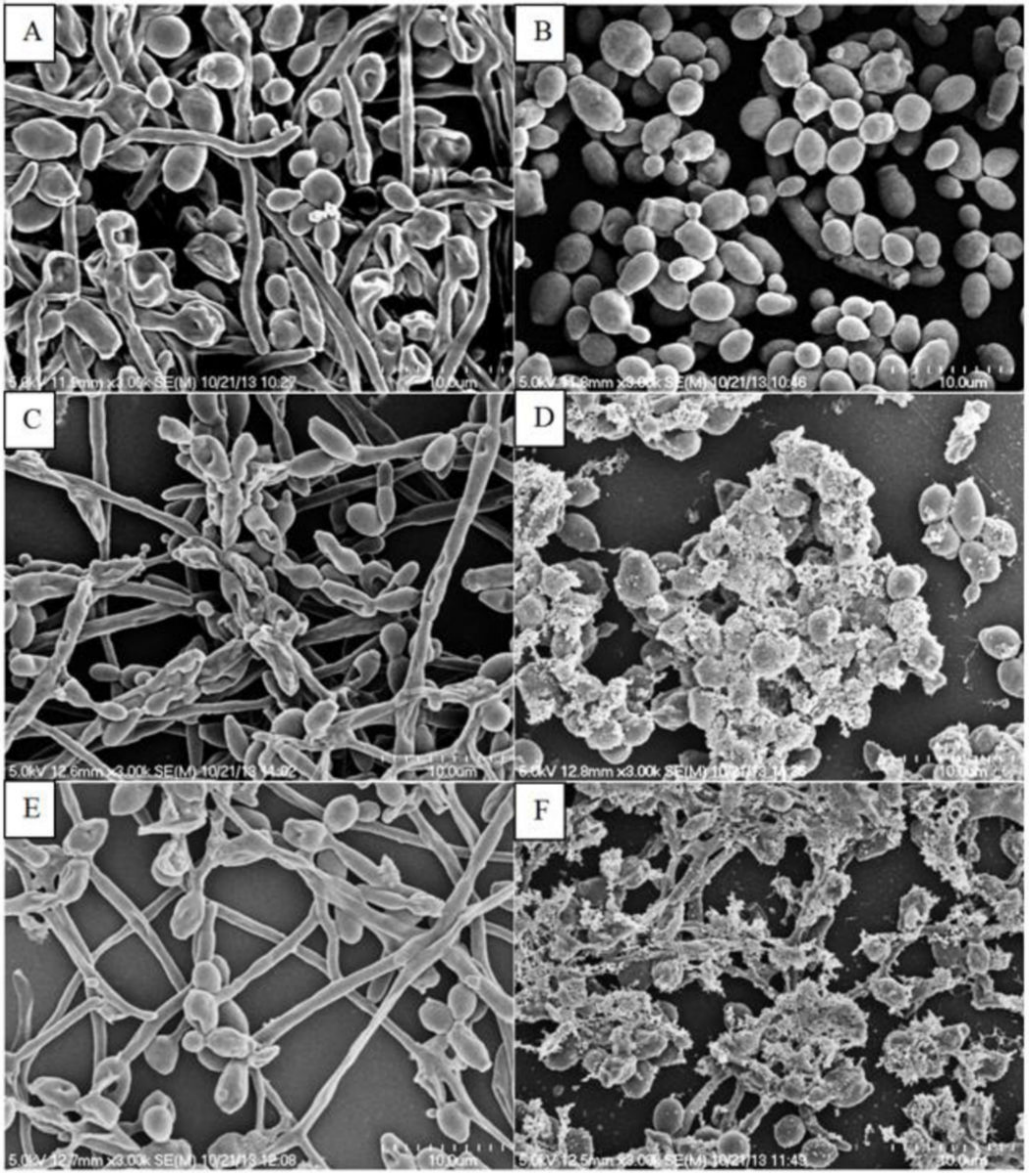
SEM images of **A)** Untreated Control; **B)** CHX treatment; **C)** 2 min treatment by 6mA plasmas; **D)** 2 min treatment by 6mA plasmas + CHX treatment; **E)** 5 min treatment by 8mA plasmas; **F)** 5 min.

As shown in (Figure 6), SEM was performed to analyse the fungal morphology changes before and after different treatments. In (Figure 6A), both yeast form and hyphae form could be observed, and the morphology of cells was intact in the untreated control samples. In contrast, only yeast form of *C. albicans* was observed after CHX treatment (Figure 6B). The SEM micrographs in (Figures 6C & 6E) show that single plasma treatments caused damages to fungal cell walls. As the plasma treatments followed by CHX treatment, the damage became increasingly more severe with massive cell wall ruptures as shown in (Figures 6D & 6F). However, most of the damaged cells were located in the superficial layer of the biofilms. There were still some intact fungal cells in the underneath cell layers in the biofilms.

## Discussion

*C. albicans* plays an important role in oral candidiasis, which is the most common fungal infection occurring in the mouth [31]. *C. albicans* biofilms are also found on the surface of implantable medical devices, including dental implants and dentures, and cause prosthesis stomatitis [1, 5]. One common preventive infection method of oral *C. albicans* biofilm formation is the use of CHX containing mouth wash [32]. However, biofilm presents CHX resistance, which hinders the efficacy of CHX [33, 34]. Depending on different test systems, the CHX concentration and exposure time also affect the efficacy of CHX in the disinfection of *C. albicans* biofilms. Our results using CHX treatment are consistent with the results by Koban *et al.*, who reported only a moderate effect on *C. albicans* biofilms by 0.1% CHX treatment for 10min [35]. Therefore, alternative approaches are needed to enhance the antifungal efficacy.

Low-temperature atmospheric plasma is increasingly claimed to have potent antibacterial activity. It has been shown to be very effective against a wide range of pathogenic bacteria and fungi [17, 18, 35, 36]. Severe damages to the cell wall and rupture of microorganisms can be caused by plasma treatment, leading to cell death [37]. Previous studies have demonstrated that low-temperature atmospheric plasma brush is effective in the inactivation of bacteria in the planktonic form and biofilm form [20, 21, 38]. These findings indicated that the low-temperature atmospheric plasma could be a promising technique in the prevention and treatment of various oral diseases. The working temperature of the plasma is less than 45°C, which indicates no heat damage from plasma heat.

This study demonstrated that low-temperature atmospheric plasma treatment could significantly disinfect *C. albicans* biofilm. MTT assay results showed that as much as 77% of *C. albicans* biofilm were inactivated after 10 min of 8mA plasma treatment. Analysis of CLSM indicated that much less viable fungal cells were observed after plasma treatment than that in the untreated control group. As seen in SEM images, plasma treatment resulted in significant morphology changes on fungal cells when compared with the untreated controls. Damages on the cell wall were noticed with the cells on the top layer of the *C. albicans* biofilms. The results from these assessing methods all showed the capability and efficacy of plasma treatment in deactivating *C. albicans* biofilms. The plasma treatment effects on *C. albicans* biofilms were also compared with CHX treatment. The results obtained in this study indicate that plasma treatment is more effective than CHX in deactivating fungal cells in biofilms. As seen in (Figure 4), the fungal reduction by CHX treatment was 33%, whereas the fungal reduction by plasma treatment was as high as 49% (for 2 min treatment by 6mA plasmas) and 70% (for 5 min treatment by 8mA plasmas). Koban *et al.* also reported similar results with their plasma systems. A log_10_ reduction factor of 6 was achieved by a plasma jet [35, 39].

It should be noted that the interaction mechanisms of plasma and CHX with bacteria are completely different. The antibacterial action of CHX is due to an increase in cellular membrane permeability followed by coagulation of the cytoplasmic macromolecules [40–42]. In contrast, the antifungal mechanism of plasma treatment is more complex than that of CHX. Plasma treatment affects not only cell walls but also other cell components, including proteins, lipids, and DNA/RNA [43]. Fungal disinfection by plasma treatment is based on reactive plasma species, including ultra-violet (UV) radiation, electronically excited neutral species, free radicals and charged particles in gas plasma [21, 37, 44]. As a result, fungal cell walls can be damaged to cause further cell lyses. Besides cell lyses, when the damage is not very extensive, Na^+^, Ca^2+^ and water can enter the cell and cause swelling, which will also cause cell death [45]. This also explains that morphological changes were observed with plasma-treated cells in SEM images, while these cells were found dead by CLSM analysis.

In this study, synergistic effects were observed with plasma treatment and CHX treatment. Fungal cell structure could be severely damaged by plasma treatment. Plasma species are capable of damaging fungal cell walls through ionic bombardment by energetic plasma particles [38]. The fungal cell walls could also be damaged through chemical etching by chemically reactive plasma species [46]. The plasma-induced fungal cell damages provide an opportunity to improve the effectiveness of antiseptics commonly used for various biofilm treatments. Plasma-induced damages on fungal cell walls could make the fungal cells become more accessible by the antimicrobial chemicals to deactivate the fungi. Therefore, a combination of plasma treatment with the commonly being used antimicrobial chemotherapy could further improve the disinfection effectiveness and efficiency for various fungal biofilms, which are usually serious challenges in clinical practice in both dental and medical fields. Furthermore, the combination approach could also shorten the plasma treatment time and therefore will be more practical in clinical applications.

A limitation in this study is the related setup of the plasma treatments. *C. albicans* biofilms grew on the bottom of a 48-well plate. During the plasma treatment, plasma brush was inserted into the wells to treat the biofilms. The relative position between the plasma flame and the biofilms was fixed with the treatment area limited to the top surfaces of the biofilms. In MTT assay, edge effects were observed with the plasma-treated groups, in which a purple ring formed at the edge of the well bottom. In other words, the fugal cells on edge were still viable because the reactive plasma species did not spread over the entire bottom area. Koban *et al.* also observed similar effects, which presented clear boundaries of damaged cells between plasma-treated area and nontreated area [35].

## Conclusion

The *in vitro* results obtained in this study conclusively demonstrated that low-temperature atmospheric plasma treatment is very effective in deactivating *C. albicans* oral biofilms. Plasma treatment exceeded the antifungal effects of the commonly used CHX treatment. Furthermore, the combination of plasma and CHX treatment increased the antifungal efficiency against *C. albicans* biofilms. The results indicate low-temperature plasma treatment provides a promising alternative to disinfect oral fungal biofilms.

## Figures and Tables

**Table 1:** Treatment conditions for each group.

Group	Treatment	Time
1	None	None
2	0.2% CHX solution	10 min
3	6 mA Plasma	2 min
4		5 min
5		10 min
6	8 mA Plasma	2min
7		5 min
8		10 min
9	6mA plasma 2 min+ 0.2% CHX 10 min	
10	8mA plasma 5 min+ 0.2% CHX 10 min	
